# The triple-isotope calibration approach: a universal and standard-free calibration approach for obtaining absolute isotope ratios of multi-isotopic elements

**DOI:** 10.1007/s00216-020-03050-4

**Published:** 2020-11-17

**Authors:** Jochen Vogl

**Affiliations:** Bundesanstalt für Materialforschung und -prüfung (BAM), Richard-Willstätter-Straße 11, 12489 Berlin, Germany

**Keywords:** Triple-isotope fractionation, Absolute isotope ratio, Mass spectrometry, Calibration, Uncertainty

## Abstract

The theory of a new calibration approach for obtaining absolute isotope ratios of multi-isotopic elements without the use of any standard has been developed. The calibration approach basically uses the difference in the instrumental isotope fractionation of two different types of mass spectrometers, leading to two different fractionation lines in a three-isotope diagram. When measuring the same sample with both mass spectrometers, the different fractionation lines have one point in common: this is the ‘true’ logarithmized isotope ratio pair of the sample. Thus, the intersection of both fractionation lines provides us with the absolute isotope ratios of the sample. This theory has been tested in practice by measuring Cd and of Pb isotope ratios in the certified reference materials BAM-I012 and NIST SRM 981 by thermal ionization mass spectrometry and by inductively coupled plasma mass spectrometry while varying the ionization conditions for both mass spectrometers. With this experiment, the theory could be verified, and absolute isotope ratios were obtained, which were metrologically compatible with the certified isotope ratios. The so-obtained absolute isotope ratios are biased by − 0.5 % in average, which should be improved with further developments of the method. This calibration approach is universal, as it can be applied to all elements with three or more isotopes and it is not limited to the type of mass spectrometers applied; it can be applied as well to secondary ion mass spectrometry or others. Additionally, this approach provides information on the fractionation process itself via the triple-isotope fractionation exponent *θ*.

**Supplementary Information:**

The online version contains supplementary material available at 10.1007/s00216-020-03050-4.

**Graphical abstract:**
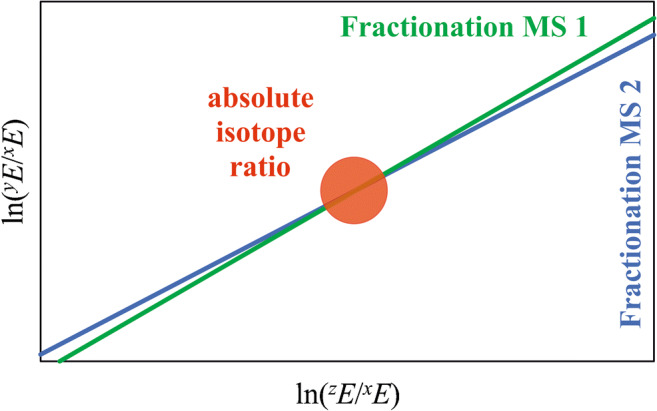
The triple-isotope calibration approach: the intersection of the triple isotope fractionation lines of an element recorded by two individual mass spectometers yields the absolute isotope ratios of this element

## Introduction

Nearly immediately after the invention of the first mass spectrograph, the isotopic composition of neon was investigated [1]. It took some decades to realize that the meanwhile developed mass spectrometers show a bias in isotope ratio measurements, the instrumental isotope fractionation (IIF, often inaccurately termed ‘mass bias’), and to find a way to correct for. A.O. Nier invented the ‘isotope mixture approach’, where highly enriched and chemically pure isotopes were mixed and the resulting mixtures together with the calculated nominal isotope ratios were used to calibrate the mass spectrometer [2]. This enabled absolute isotope ratio measurements and revolutionized the determination of atomic weights in the following decades. Within the past two decades, the major deficiencies of this approach have been solved, which are the iterative solution and the use of fractionation models to transfer a calibration from one isotope ratio to a non-calibrated isotope ratio. It was demonstrated recently for magnesium that the isotope mixture approach can be applied as an ab initio calibration without any a priori assumptions, leading to absolute isotope ratios with measurement uncertainties of around 0.01 % [3]. A modification of this approach, which mainly has been used in thermal ionization mass spectrometry (TIMS) is the double-spike technique, where two enriched isotopes of a multi-isotopic element were added to the sample and were used to calibrate the isotope ratio measurements [4]. Drawbacks for obtaining absolute isotope ratios are the application of fractionation models and the required accurate quantification of the element amount in the sample and the double-spike. Additionally, this technique can be applied only to elements with at least four isotopes; with the addition of a second blend also elements with three isotopes are accessible [5]. A completely different approach in TIMS is the total evaporation technique, which has been developed for the measurement of U and Pu first. In TIMS the IIF progresses over the duration of the measurement [6]. When integrating the ion beam of the isotopes of interest over the whole duration of a measurement, i.e. from the first measurable signal until the analyte reservoir is exhausted, fractionation takes no effect and the ratio of the integrated ion beams gives the absolute isotope ratio. In multi-collector inductively coupled plasma mass spectrometry (MC-ICP-MS), a completely different approach, the ‘mass bias regression model’, has been added [7]. This technique is based on the observed correlated drift in isotope ratios of two elements occurring in MC-ICP-MS, whereby one element is the analyte element of interest and the other element is the reference element represented by a standard. All available methods for correcting IIF to obtain absolute isotope ratios require a standard, represented either by an isotopic certified reference material (iCRM) as in the case of the ‘regression mass bias model’ or by specifically prepared solutions of enriched isotopes as in the case of the ‘isotope mixture approach’ and the ‘double-spike technique’. Even in total evaporation TIMS, a standard is used for correction when the residual bias should be reduced to < 0.05 %. Thus, all correction techniques refer to a standard in one way or another. Additionally, total evaporation TIMS is limited to a few elements only. The ‘isotope mixture approach’ and the ‘double-spike technique’ heavily depend on the availability of the enriched isotopes. Therefore, alternative approaches for correcting IIF are urgently needed, especially such, which do not need standards, and which are applicable more widely. Exactly this is provided by the new triple-isotope calibration approach presented here, which utilizes the instrumental isotope fractionation behaviour for its own correction.

## Theoretical background of the applied approach

When elements undergo a chemical or a physical process leading from a starting material (A) to a product (B), most likely their isotopes are being treated disproportionally, which results in the so-called isotope fractionation. For any of these processes, the extent of this fractionation can be described by the isotope fractionation factor *α* (Eq. 1), with *n*(^*i*^*E*) being the amount of substance of isotope *i* and *n*(^*j*^*E*)/*n*(^*i*^*E*) representing the isotope ratio *R*^*j*/*i*^.

1 $$ {\alpha}_{A-B}^{j/i}=\frac{{\left(\frac{n\left(j,E\right)}{n\left(i,E\right)}\right)}_{\mathrm{B}}}{{\left(\frac{n\left(j,E\right)}{n\left(i,E\right)}\right)}_{\mathrm{A}}} $$

For elements with three (or more) isotopes x, y, z sorted in ascending mass, the mass dependence of the isotope fractionation yields correlated isotope ratios. The isotope fractionation factors of the isotope ratios *R*^*y*/*x*^ and *R*^*z*/*x*^ are scaled by the ‘triple-isotope fractionation exponent’ *θ* (Eq. 2) [8].

2 $$ {\alpha}_{\mathrm{A}\hbox{-} \mathrm{B}}^{y/x}={\left({\alpha}_{\mathrm{A}\hbox{-} \mathrm{B}}^{z/x}\right)}^{\theta } $$

3 $$ {\theta}_{\mathrm{A}\hbox{-} \mathrm{B}}=\frac{\ln {\left({R}^{y/x}\right)}_{\mathrm{B}}-\ln {\left({R}^{y/x}\right)}_{\mathrm{A}}}{\ln {\left({R}^{z/x}\right)}_{\mathrm{B}}-\ln {\left({R}^{z/x}\right)}_{\mathrm{A}}} $$

**Fig. 1:**
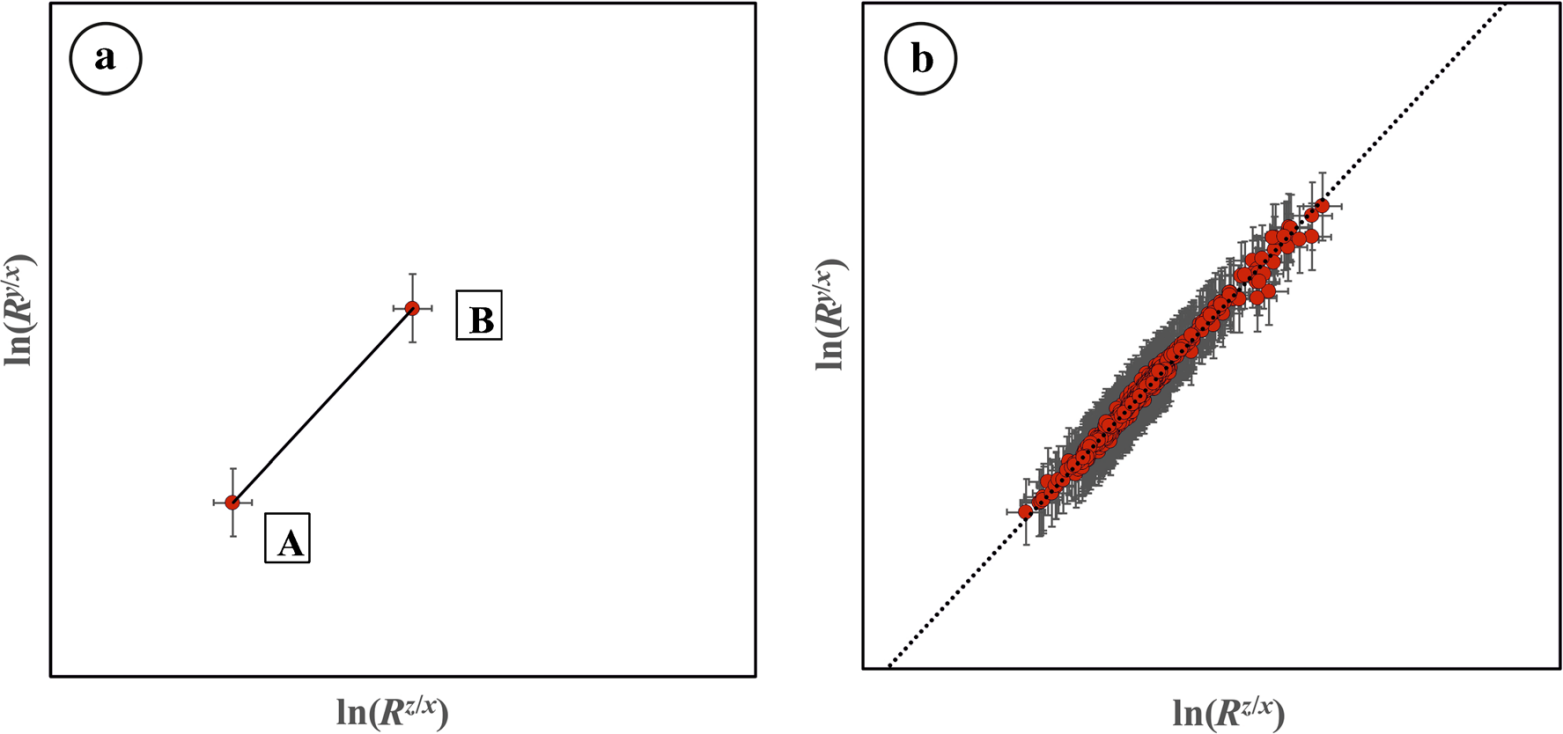
Three-isotope diagrams for an isotope fractionation process, where (**a**) starting material and product are known and (**b**) starting material is unknown, but several products of the same fractionation process are accessible. The lines represent the triple-isotope fractionation line with *θ* as slope

*θ* describes the nature of the isotope fractionation, i.e. whether it is an equilibrium or a non-equilibrium process. It can be described as the slope in the three-isotope diagram (Fig. 1a), where either the natural logarithm of the isotope ratios according to Eq. 3 or linearized isotope delta values are plotted [9].

In most cases, however, the starting material (A) is not accessible. Then, samples which underwent the same fractionation process, but to a varying extent, can be plotted in the three-isotope diagram (Fig. 1b). The starting material will be located somewhere on the regression line and the slope provides the apparent *θ* value. This is applicable to natural processes, but as well to technical ones, such as the isotope fractionation in a mass spectrometer. Carefully changing the ionization conditions in the ion source of a mass spectrometer should give systematic isotope ratio variations similar to Fig. 1b, with the ‘true’ isotope ratios (the isotope ratios of the starting material) being somewhere on the regression line. When the same sample is now measured with another mass spectrometer featuring a different ion source or simply offering a different fractionation process, a second set of isotope ratios will be obtained yielding a regression line with a different slope, which is not parallel to the regression line of the first dataset. As there is only one pair of ‘true’ isotope ratios for both measurements, the intersection of both regression lines will give the ‘true’ logarithmized isotope ratios. The mathematical background is provided in the Electronic Supplementary Material (ESM_1), section 1.

## Materials and methods

For all sample solution preparations and subsequent dilutions, only precleaned labware and high-purity water and acids were used. Further details can be obtained from the literature [10]. For all isotope ratio measurements within this study, the iCRMs BAM-I012 [11] and NIST SRM 981 [12] were used as samples.

The MC-ICP-MS measurements were carried out in standard configuration using a Neptune Plus type (Thermo Scientific, Bremen, DE) instrument. Different degrees of the instrumental isotope fractionation were obtained by changing the plasma power from 1100 to 1300 W in steps of 20 W each and by changing the nebulizer gas flow from 0.01 mL/min below to 0.01 mL/min above the optimum set value by steps of 0.005 mL/min. For each of the steps, one blank measurement and five isotope ratio measurements of the iCRMs were carried out. Amplifier gain and background calibration was carried before each daily sequence. Each measurement was corrected for the blank measured in dilute nitric acid (*w* = 20 g/kg).

MC-TIMS measurements were carried out using a Sector 54 (Micromass, Cheshire, UK) instrument. Cd and Pb solutions were diluted such that 1 μL of each solution could be loaded directly on Re single filaments using the silica gel technique. Different degrees of the instrumental isotope fractionation were achieved by variations in the loaded analyte mass—5 ng, 15 ng, 50 ng, 250 ng and 1000 ng for Cd and 5 ng, 10 ng, 50 ng, 250 ng and 1000 ng for Pb—and in the target intensity, which was varied from 220 mV to 1020 mV for ^114^Cd and from 600 mV to 1400 mV for ^208^Pb in steps of 100 mV. For each setting, at least two filaments were loaded and measured, with 12 blocks each and 25 measurement cycles (4 s integration time) per block. Background corrections were carried out for each block and gain factors, which were obtained by daily amplifier gain calibration, were applied. Block data, i.e. averages of 25 cycles, were used as individual measurement. The instrument parameters and the measured isotope ratios were provided as ESM_1 and ESM_2.

## Results and discussion

**Fig. 2:**
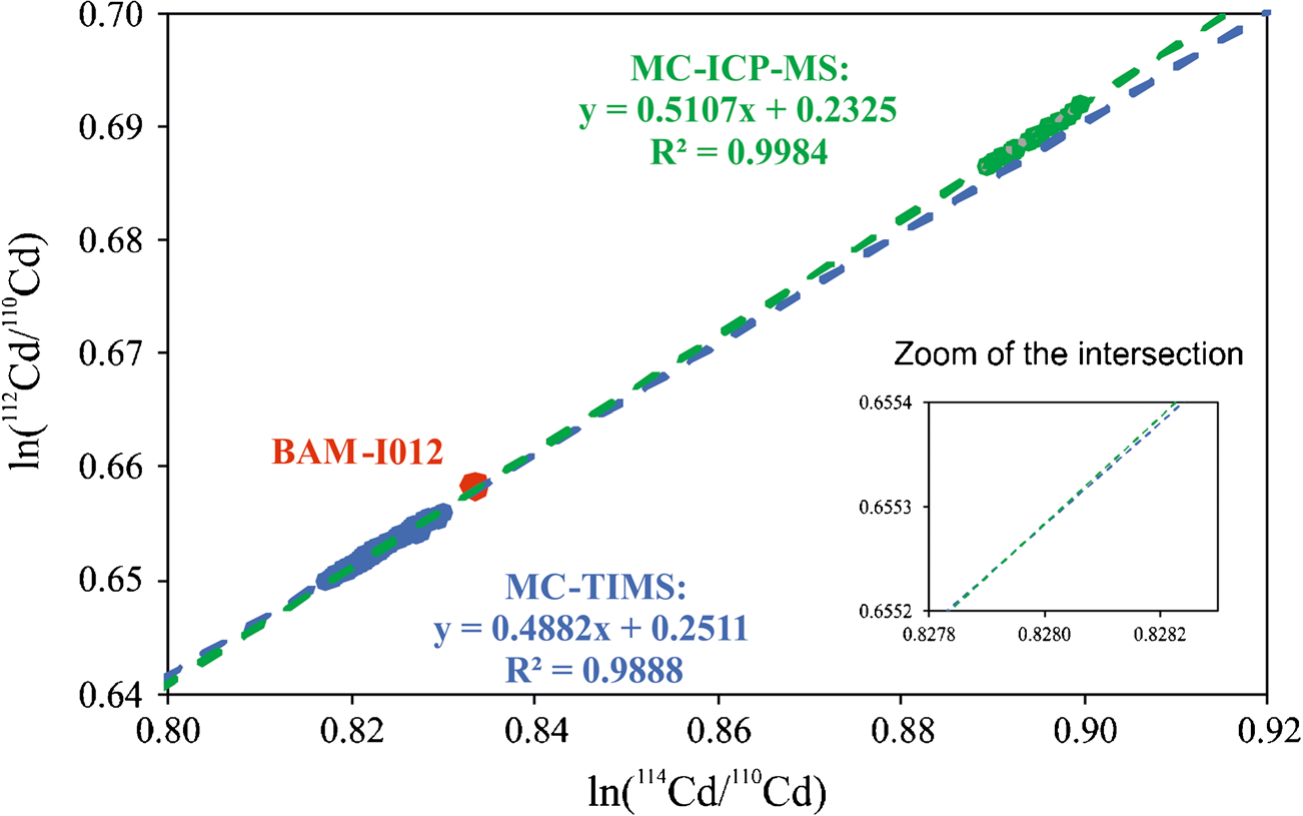
Three-isotope diagram for Cd: ln(^112^Cd/^110^Cd) vs. ln(^114^Cd/^110^Cd); the blue dots depict the MC-TIMS data, the green dots depict the MC-ICP-MS data and the red dot shows the certified values of BAM-I012 (uncertainty bars are not visible)

The above outlined model assumption was tested by applying MC-TIMS and MC-ICP-MS. The completely different ion sources ensure different fractionation processes. Variations in the analyte mass loaded onto the filaments and the target intensity in MC-TIMS should provide a distinct IIF line in a three-isotope plot. Variations in the plasma power and the nebulizer gas flow in MC-ICP-MS should provide another distinct IIF line in the same three-isotope plot with the intersection of both IIF lines giving the absolute isotope ratio of the sample. This experiment was carried out for Cd and Pb isotope ratio measurements using the iCRMs BAM-I012 (Cd) and NIST SRM 981 (Pb), because they provide certified absolute isotope ratios, which can be used as reference. The raw isotope ratios (only background/blank and gain corrected) were plotted in a three-isotope diagram as described above and visualized in Fig. 2 for the example of ln(^112^Cd/^110^Cd) vs. ln(^114^Cd/^110^Cd)—short notation 112-114-110.

Both IIF lines show very similar slopes and it is visible that the intersect is close to, but not exactly at, the corresponding ln values of the certified BAM-I012 ratios. The correlation coefficient of the MC-ICP-MS line is closer to 1 than that of the MC-TIMS line, demonstrating a higher precision in the isotope ratio measurement for MC-ICP-MS and the fact that with the chosen parameters the IIF is more under control than for TIMS.

Similar diagrams have been obtained for cadmium 111-113-110 and 112-114-111 and for Pb 207-208-206. It has to be noted that the MC-TIMS data in the cadmium 111-113-110 diagram are distributed in elliptic form with some outliers. For the other three-isotope diagrams, i.e. cadmium 112-114-110, cadmium 112-114-111 and lead 207-208-206, linear distributions were obtained. Each diagram provides an intersect of the two fractionation lines. The isotope ratios of Cd and Pb obtained from the delogarithmized intersects are displayed in Table 1, together with their combined measurement uncertainties, *u*_c_; the reference values; the bias to the reference values and the *E*_n_ value. The latter is the difference between the measured value and the reference value divided by the expanded uncertainty of this difference (see ESM_1, eqn. 13 to 16). It depicts the metrological compatibility between both values within the stated expanded uncertainties (*k* = 2) [13]. For *E*_n_ values of less than or equal to 1, both values are metrological compatible with each other; in older terms, they agree within their uncertainties.

**Table 1:** Obtained absolute Cd and Pb isotope ratios for BAM-I012 and NIST SRM 981 together with their combined measurement uncertainties, *u*_c_; the reference values; the bias to the reference values and the *E*_n_ values

Element	Diagram	Quantity	Isotope ratio (mol mol^−1^)				Bias (%)	*E*_n_
			Measured value	*u*_c_	Reference value	*u*_c_		
Cd	111-113-110	*n*(^111^Cd)/*n*(^110^Cd)	1.0230	0.0017	1.025599	0.000055	− 0.3	0.773
Cd	111-113-110	*n*(^113^Cd)/*n*(^110^Cd)	0.9717	0.0020	0.979232	0.000096	− 0.8	1.888
Cd	112-114-110	*n*(^112^Cd)/*n*(^110^Cd)	1.926	0.022	1.93172	0.00023	− 0.3	0.138
Cd	112-114-110	*n*(^114^Cd)/*n*(^110^Cd)	2.289	0.027	2.30114	0.00038	− 0.5	0.236
Cd	112-114-111	*n*(^112^Cd)/*n*(^111^Cd)	1.877	0.093	1.88352	0.00016	− 0.4	0.037
Cd	112-114-111	*n*(^114^Cd)/*n*(^111^Cd)	2.222	0.139	2.24371	0.00031	− 1.0	0.079
Pb	207-208-206	*n*(^207^Pb)/*n*(^206^Pb)	0.9125	0.0020	0.91464	0.00017	− 0.2	0.542
Pb	207-208-206	*n*(^208^Pb)/*n*(^206^Pb)	2.156	0.039	2.1681	0.0004	− 0.6	0.155

The bias between the obtained absolute isotope ratios and the certified ones is in the range of − 0.2 % to − 1 %. Although not completely satisfying, concerning todays requirements, the bias is covered by the measurement uncertainty and thus all obtained absolute isotope ratios are metrologically compatible with the certified values of the iCRMs, except for the *n*(^113^Cd)/*n*(^110^Cd) isotope ratio. This agreement demonstrates the validity of the postulated calibration approach within the stated measurement uncertainties. An investigation of the remaining systematic negative bias’s origin or even an accurate determination of its nature is hardly possible, because the bias is fully covered by the measurement uncertainty. However, two possible scenarios can be assumed: (a) As the intersection of the TIMS and the ICP-MS regression lines is always at or close to the measured TIMS bulk data (at least closer than to the ICPMS bulk data) and as, due to the specific instrumental isotope fractionation, TIMS raw data are always lower than the certified ratios while ICP-MS raw data are always higher (for isotope ratios heavy over light isotope), the less precise TIMS data (higher standard deviation, smaller coefficient of determination *R*^2^) may lead to negatively biased results, and (b) Mass-independent fractionation effects (or other artefacts) may be responsible for the bias, although they are rather unlikely to occur at this level and have not yet been observed to this extent. A proof for either of the two assumptions is not possible at this stage and will require further investigations and a further improvement of this method.

Certainly, at this stage, the obtained measurement uncertainties and the residual bias do not fulfil todays requirements for absolute isotope ratios, and at the current state, this approach is not comparable to the ‘isotope mixture approach’ [3] and the ‘mass bias regression model’ [7] in terms of measurement uncertainties, but it offers a valid alternative when no iCRM and no enriched isotopes are available. For wider applications, further development of this calibration approach is required.

When changing the axis in the cadmium 112-114-110 diagram to obtain the 114-112-110 diagram, the bias in the *n*(^112^Cd)/*n*(^110^Cd) and the *n*(^114^Cd)/*n*(^110^Cd) isotope ratios is being reduced to 0.3 % and 0.05 % respectively. This points to a non-linear artefact and requires consideration in the further development. In the other diagrams, there are subtle differences in the bias when swapping the axis, but not at this level.

Additional information is obtained for both mass spectrometers in the form of the triple-isotope fractionation exponent *θ*, which describes the isotope fractionation within the mass spectrometer. Considering that fractionation in magnetic sector fields and Faraday cup detectors is comparatively low, it describes in fact the fractionation in the ion source and the interface. When looking at the *θ* values of these measurements (Table 2), it is obvious that all *θ* values obtained by MC-ICP-MS measurements overlap (within expanded uncertainties) with the theoretical range of equilibrium and non-equilibrium isotope fractionation, while *θ* values obtained by MC-TIMS in most cases do not. For TIMS, this might be an indication for mass-independent effects, whereby it is more likely that two or more mass-dependent fractionation effects are coupled, which can lead to *θ* values outside the theoretical range [13]. In the case of MC-ICP-MS, it is a strong indication for IIF, which is dominated by a mass-dependent process.

**Table 2:** Obtained *θ* values for MC-TIMS and MC-ICP-MS with their associated measurement uncertainties and the theoretical *θ* values of equilibrium and non-equilibrium isotope fractionation

Element	Diagram	MS	*θ*	*U*^a^	Theoretical *θ*^b^		Within range
					non-eq	eq	
Cd	111-113-110	TIMS	0.574	0.074	0.337	0.340	−
Cd	111-113-110	ICPMS	0.341	0.007	0.337	0.340	+
Cd	112-114-110	TIMS	0.488	0.005	0.504	0.509	−
Cd	112-114-110	ICPMS	0.511	0.005	0.504	0.509	+
Cd	112-114-111	TIMS	0.632	0.012	0.336	0.339	−
Cd	112-114-111	ICPMS	0.339	0.003	0.336	0.339	+
Pb	207-208-206	TIMS	0.445	0.016	0.501	0.503	+
Pb	207-208-206	ICPMS	0.498	0.003	0.501	0.503	+

^a^Expanded measurement uncertainty with *U* = *k* · *u*_c_ and *k* = 2

^b^For calculation of theoretical *θ* values, see ref. [9] or the ESM_1, eqn. 17 and 18

## Outlook

The here presented standard-free triple-isotope calibration approach is valid. Admittedly, the obtained measurement uncertainties do not meet the requirements for many isotope ratio applications. Therefore, the procedure needs to be improved, mainly on the TIMS side. Here, the range within which the parameters were varied can be tightened to produce less outlier values, especially the very low analyte masses of 5 ng loaded onto the filaments should be avoided, because in these cases, the analyte reservoir is quickly exhausted leading to extreme fractionation behaviours. Variations in the target intensity, which affect the filament temperature via the filament current, might be reduced as well to keep the experiments under better controlled conditions. In MC-ICP-MS, the range of the tested plasma power might be reduced a bit, as the 1300 W stage produced outliers at least for Pb. The triple-isotope calibration approach is not limited to TIMS and ICP-MS; it might be applied to other mass spectrometers as well such as SIMS together with ICP-MS. As long as different ionization conditions can be realized, i.e. two different *θ* values, it might be even applicable to two different instruments of the same type of mass spectrometer, e.g. two MC-ICP-MS instruments.

With the obtained TIMS *θ* values in mind, it might be interesting as well to decouple the evaporation and ionization process by applying double-filament techniques. When varying the evaporation and ionization filaments independently, it might be possible to obtain new information on the ionization process in TIMS and to check whether it is a two- or more-stage process.

## Supplementary information

ESM 1 (PDF 284 kb)

ESM 2 (XLSX 251 kb)
