# Supplementary material for: The triple-isotope calibration approach: a universal and standard-free calibration approach for obtaining absolute isotope ratios of multi-isotopic elements
Source: Anal Bioanal Chem. 2020 Nov 17;413(3):821–6. doi: 10.1007/s00216-020-03050-4 (PMC7808992; doi:10.1007/s00216-020-03050-4)
Supplement: Supplementary file 1 — (PDF 284 kb) [file 216_2020_3050_MOESM1_ESM.pdf]

Analytical and Bioanalytical Chemistry

Electronic Supplementary Material

The triple-isotope calibration approach: a universal and standard-free calibration approach for obtaining absolute isotope ratios of multi-isotopic elements

Jochen Vogl

Additional file available under “Supplementary material”.

Table S1 Operating conditions for the applied MC-TIMS

Parameter	MC-TIMS
Instrument type	Sector 54, Micromass
Autosampler	Sample turret with 20 filaments
Filaments	Re single filaments on glass beads
Loading technique	Silica gel technique
Loaded analyte mass	5 ng to 1000 ng Cd or Pb
Mass resolution mode	Low
Measured isotopes	^{110}Cd, ^{111}Cd, ^{112}Cd, ^{113}Cd, ^{114}Cd<br>^{206}Pb, ^{207}Pb, ^{208}Pb
Faraday detectors	L1, C, H1, H2, H3 (Cd)<br>C, H1, H2 (Pb)
Gain calibration	Before each sequence
Baseline measurement	Before each filament
Amplifier resistors	10^{11} Ω
Integration time	4 s
Blocks / cycles	12 / 25
Signal intensity	220 mV to 1020 mV (^{114}Cd)<br>600 mV to 1400 mV (^{208}Pb)
Typical isotope ratios repeatability (s_{rel}) [b]	< 0.05 %

[b] Relative standard deviation s_{rel} within one measurement

Table S2 Operating conditions for the applied MC-ICP-MS

Parameter	MC-ICP-MS
Instrument type	Neptune Plus, Thermo Scientific
Autosampler	ESI SC2 DX
Aspiration mode	Self-aspirating
Nebulizer	PFA 100 µL min^{-1}
Spray chamber	Combined cyclonic & Scott (quartz)
Interface	Jet
Cones	Ni sampler & Ni H skimmer
Cool gas flow rate	16 L min^{-1}
Auxiliary gas flow rate	0.8 L min^{-1}
Sample gas flow rate	1.08 - 1.12 L min^{-1}
RF power	1100 W to 1300 W
Guard electrode	On
Mass resolution mode	Low
Measured isotopes	^{110}Cd, ^{111}Cd, ^{112}Cd, ^{113}Cd, ^{114}Cd<br>^{206}Pb, ^{207}Pb, ^{208}Pb
Faraday detectors	L2, L1, C, H1, H2 (Cd)<br>C, H1, H2 (Pb)
Gain calibration	Before each sequence
Baseline measurement	Before each sequence
Amplifier resistors	10^{11} Ω
Integration time	4.194 s
Blocks / cycles	1 / 50
Sensitivity [a]	31 $V \cdot mg^{-1} \cdot kg$ (Cd)<br>96 $V \cdot mg^{-1} \cdot kg$ (Pb)
Mass fractions of solutions used	0.3 mg kg^{-1} (Cd)<br>0.1 mg kg^{-1} (Pb)
Typical blank intensity	< 0.1 mV on ^{114}Cd<br>< 0.5 mV on ^{208}Pb
Typical isotope ratio repeatability (s_{rel}) [b]	< 0.018 % for Cd<br>< 0.006 % for Pb

[a] Sum of all ion intensities of an element per 1 mg kg^{-1} element in the solution

[b] Relative standard deviation s_{rel} within one measurement

1. Mathematics for the triple isotope calibration approach

1.1 Equation system

In the following section it is explained how the absolute isotope ratios are obtained using the triple isotope calibration approach:

1) The measured isotope ratios $r_i^{y/x}$ and $r_i^{z/x}$ of element E with the isotope masses x < y < z are recorded with two mass spectrometers MS1 and MS2, with *i* =MS1, MS2.
2) Of all measured isotope ratios $r_i^{y/x}$ and $r_i^{z/x}$ the natural logarithm was taken yielding both $r_i^{y/x'}$ and $r_i^{z/x'}$:

   $r_i^{y/x'} = \ln(r_i^{y/x})$ eqn. 1

   $r_i^{z/x'} = \ln(r_i^{z/x})$ eqn. 2
3) All data $r_i^{y/x'}$ and $r_i^{z/x'}$ are plotted in the triple isotope diagram and linear regression is performed for MS1 data and MS2 data separately yielding two regression lines with the following equations:

   $y_{\mathrm{MS1}} = a_{\mathrm{MS1}} \cdot x_{\mathrm{MS1}} + b_{\mathrm{MS1}}$ eqn. 3

   $y_{\mathrm{MS2}} = a_{\mathrm{MS2}} \cdot x_{\mathrm{MS2}} + b_{\mathrm{MS2}}$ eqn. 4

   a_{MS1} and b_{MS1} were obtained from the linear regression for MS1; a_{MS2} and b_{MS2} were obtained from the linear regression for MS2.
4) The intersection (IS) of both regression lines is obtained by equating:

   $y_{\mathrm{MS1}} = y_{\mathrm{MS2}} = y_{\mathrm{IS}}$ eqn. 5

   This gives: $a_{\mathrm{MS1}} \cdot x_{\mathrm{MS1}} + b_{\mathrm{MS1}} = a_{\mathrm{MS2}} \cdot x_{\mathrm{MS2}} + b_{\mathrm{MS2}}$ eqn. 6

   The intersection gives: $x_{\mathrm{MS1}} = x_{\mathrm{MS2}} = x_{\mathrm{IS}}$ eqn. 7

   which results in: $x_{\mathrm{IS}} = -\frac{(b_{\mathrm{MS2}} - b_{\mathrm{MS1}})}{(a_{\mathrm{MS2}} - a_{\mathrm{MS1}})}$ eqn. 8

   x_{IS} is inserted in eqn. 3 or 4 yielding eqn. 9 and 10, respectively:

   $y_{\mathrm{IS}} = a_{\mathrm{MS1}} \cdot x_{\mathrm{IS}} + b_{\mathrm{MS1}}$ eqn. 9

   $y_{\mathrm{IS}} = a_{\mathrm{MS2}} \cdot x_{\mathrm{IS}} + b_{\mathrm{MS2}}$ eqn. 10
5) x_{IS} and y_{IS} are then delogarithmized, and then yield the absolute isotope ratios $R^{y/x}$ and $R^{z/x}$ in the sample:

   $R^{y/x} = e^{y_{\mathrm{IS}}}$ eqn. 11

   $R^{z/x} = e^{x_{\mathrm{IS}}}$ eqn. 12

1.2 Calculation of the measurement uncertainty

The linear regression was carried out in Excel® by applying the LINEST function. This function calculates the linear regression based on the least squares method and gives the parameters *a* (slope) and *b* (intercept), as well as the variances of *a* and *b*.

These variances are then used to calculate the measurement uncertainty of the absolute ratios $R^{y/x}$ and $R^{z/x}$ in GUM Workbench (Metrodata GmbH, Braunschweig, Germany) via eqn. 8 to 12.

2. Equations for the E_n value

The E_n value is used for the assessment whether or not two data are metrologically compatible, in other words whether or not they agree within their stated uncertainties. The mathematical background is presented in eqn. 13 to 16. Consequentially, two values are metrologically compatible, when their associated E_n value is ≤ 1.

$$d_{ij} = x_i - x_j \qquad \text{eqn. 13}$$

$$u^2\left(d_{ij}\right) = u^2\left(x_i\right) + u^2\left(x_j\right) - 2 \times \mathrm{cov}\left(x_i, x_j\right) \qquad \text{eqn. 14}$$

$$U\left(d_{ij}\right) = k \cdot u\left(d_{ij}\right) \qquad \text{with } k = 2 \qquad \text{eqn. 15}$$

$$E_\mathrm{n} = \frac{\left|d_{ij}\right|}{U\left(d_{ij}\right)} \qquad \text{eqn. 16}$$

3. Equations for the theoretical *θ* values

θ is a property intrinsic to mass-dependent isotope fractionation, and differs for equilibrium and non-equilibrium reactions. $\theta_{\mathrm{equilibrium}}$ can be predicted from the quantum mechanical behaviour of isotopes (eqn. 17; equation 15 in Young et al. 2002 [1]):

[1] Young E. D., Galy A. and Nagahara H. (2002) Kinetic and equilibrium mass-dependant isotope fractionation laws in nature and their geochemical and cosmochemical significance. Geochim. Cosmochim. Acta 66, 1095–1104.

$$\theta_{\text{equilibrium}} = \frac{\left(\frac{1}{m_x} - \frac{1}{m_y}\right)}{\left(\frac{1}{m_x} - \frac{1}{m_z}\right)} \qquad \text{eqn. 17}$$

$\theta_{\text{non-equilibrium}}$ can be predicted from calculations of the motions of effective masses (equation 21 in Young et al. 2002 [1]).

$$\theta_{\text{non-equilibrium}} = \frac{\ln\left(\frac{m'_x}{m'_y}\right)}{\ln\left(\frac{m'_x}{m'_z}\right)} \qquad \text{eqn. 18}$$

Note: *m* in eqn. 17 represents the masses of the isotopes, while *m'* in eqn. 18 represents the masses of the isotopologues.
